# Association between echogenicity of ascites and its classification in dogs

**DOI:** 10.3389/fvets.2026.1832745

**Published:** 2026-08-06

**Authors:** Silvia Biondo, Samuel Jakovljevic, Mireia Pascual, Maria Fernandez, Ben Fayers, Ferran Valls Sanchez

**Affiliations:** 1 Dick White Referrals, Part of Linnaeus Veterinary Ltd, Cambridgeshire, United Kingdom; 2 Veterinaria del Mar, Barcelona, Spain; 3 Southfields Veterinary Specialists, Essex, United Kingdom; 4 WeYouVets, Essex, United Kingdom

**Keywords:** ascites, cytology, dog, exudate, peritoneal effusion, ultrasound

## Abstract

**Background:**

Ascites in dogs results from a range of systemic and localized disorders. Early distinction between transudative and exudative peritoneal effusions, and between septic and non-septic exudates, is crucial for guiding emergency interventions and treatment strategies.

**Objective:**

To evaluate the relationship between echogenicity of ascites and results of fluid analysis, and to determine whether echogenicity can help differentiate transudates from exudates.

**Methods:**

Retrospective study including 100 client-owned dogs with ultrasonographically confirmed peritoneal effusion and fluid analysis performed within 3 h of imaging. Effusions were classified according to total nucleated cell count (TNCC) and total protein concentration (TPC) as follows: transudates (<1,500 cells/μL, <2.5 g/dl), exudates (≥5,000 cells/μL, ≥2.5 g/dl), or other (hemoperitoneum, biliary peritonitis, uroperitoneum). Static DICOM images and cine loops were acquired and independently assessed by a board-certified radiologist and a radiology resident. Consensus was reached for all cases. Effusions with uniform echo-free appearance were deemed anechogenic; those containing multiple or confluent hyperechoic foci were classified as echogenic.

**Results:**

Sixty-six effusions were echogenic and 34 anechogenic. Of the 40 exudates, 80% were echogenic compared to 43% of the 41 transudates. All 17 septic exudates (100%) were echogenic compared to 65% of 23 non-septic exudates. TNCC (≥5,000 cells/μL) and TPC (>4.5 g/dL) were strongly correlated with echogenic appearance.

**Conclusions:**

Echogenicity correlated significantly with exudative and septic effusions. Echogenicity may offer a rapid, non-invasive method to complement cytologic and biochemical analyses. Incorporating echogenicity assessment into abdominal ultrasonographic protocols may hasten critical clinical decision-making and improve outcomes in dogs with ascites.

## Introduction

Ascites is characterized by the accumulation of serous fluid in the peritoneal cavity and represents a significant clinical observation in dogs. The formation of ascites may arise from localized conditions within the peritoneal cavity or systemic disorders that influence fluid balance (1). The conventional classification of a peritoneal effusion relies on its appearance, total nucleated cell count (TNCC), and total protein concentration (TPC). Cytological examination and supplementary biochemical analyses are performed to refine fluid classification (2, 3). Based on TNCC and TPC, peritoneal effusions are categorized into three primary types: protein-poor transudate, protein-rich transudate (historically referred to as modified transudates) and exudate (3). Transudates typically develop due to elevated hydrostatic pressure or diminished oncotic pressure and are often associated with systemic conditions such as congestive heart failure, portal hypertension, and hypoalbuminemia (2). In contrast, exudates are distinguished by their high protein content and cell count (1, 3) and generally arise in response to infections, inflammation, or neoplasia (3). Carrying out biochemical and cytological examinations of peritoneal fluid is essential for ascertaining the precise nature of the effusion (3).

Accurate classification of peritoneal fluid types in dogs is crucial for informing clinical decision-making. Distinguishing between septic and non-septic exudates presents one of the most challenging scenarios in clinical practice (2, 3). The identification of intracellular bacteria through cytological analysis is recognized as the gold standard for diagnosing septic peritonitis (3). However, this approach shows a sensitivity of only 63% and a specificity of 89.6% (3). The primary difficulty lies in the moderate sensitivity of cytological analysis, which depends heavily on the operator's expertise in accurately detecting intracellular bacteria in peritoneal fluid. Failure to identify bacteria may impede or misguide critical treatment decisions in emergencies, where rapid and precise differentiation between septic and non-septic exudates is vital (3). Other laboratory parameters, such as fluid glucose and lactate measurements, have been assessed but have not demonstrated enhanced diagnostic efficacy (4).

Ultrasonography is a widely used imaging technique in the evaluation of dogs presenting with peritoneal effusion. It provides a non-invasive, safe, and readily accessible method to obtain both qualitative and quantitative information about the effusion (5–8). Furthermore, ultrasonography is routinely employed to guide abdominocentesis. There is consensus that the echogenicity of abdominal fluid is influenced by several factors, including operator dependence, variable equipment settings, and overlapping sonographic characteristics among different fluid types. These challenges restrict the ability to characterize fluid solely based on echogenicity (7, 8). Ultrasonographic findings should be correlated with the patient's clinical history and substantiated through additional imaging or fluid analysis. In human medicine, quantitative B-mode gray-scale ultrasonographic histograms and Doppler techniques have been explored to differentiate types of ascites; however, these methods remain investigational and have not attained widespread clinical adoption (9–11).

This study aims to investigate the correlation between ultrasonographic echogenicity of peritoneal effusions and fluid analysis in dogs. It is hypothesized that transudates will exhibit lower echogenicity due to their reduced particle and cellular content. Conversely, exudates will demonstrate higher echogenicity due to their increased protein, cellular, and inflammatory cell content.

## Materials and methods

A retrospective study was conducted at a single veterinary referral hospital [Dick White Referrals (DWR), Six Mile Bottom, Cambridgeshire, United Kingdom] on 100 dogs admitted between October 2019 and March 2024. The inclusion criteria for the study were dogs that had undergone abdominal ultrasonography and had a documented peritoneal effusion, with fluid analysis results available from samples collected within 3 h of the ultrasonographic examination.

The analysis of peritoneal fluid included TNCC, TP, and cytological examination. This testing was performed at the DWR Diagnostic Lab (Antech Diagnostics Limited, ISO 900: 2015). Fluid classification adhered to established guidelines: effusions were classified as exudates if the TPC exceeded 2.5 g/dL and the TNCC was at least 5,000 cells/mL (1–3). Septic effusions were identified through cytological examination, which revealed intracellular bacteria, combined with fluid characteristics typical of exudates, such as a TPC exceeding 2.5 g/dL and a TNCC of at least 5,000 cells/mL, confirming an infectious etiology. Conversely, effusions were classified as transudates if the TPC was below 2.5 g/dL and the TNCC was below 1,500 cells/mL. For the purposes of this study, both protein-poor and protein-rich transudates were grouped together under the designation transudates. Hemoabdomen, bile peritonitis and Uroabdomen were consolidated into a single category labeled “Other.” Hemoabdomen was diagnosed when the peritoneal fluid showed a hematocrit (packed cell volume; PCV) above 3% and clear evidence of hemorrhage, such as a high number of erythrocytes. Bile peritonitis was diagnosed when the peritoneal fluid displayed a distinctive greenish-yellow coloration, with biochemical analysis indicating bilirubin levels 2:1 relative to serum and a high bile acid concentration exceeding 200 μmol/L. Uroabdomen was identified based on fluid analysis revealing creatinine concentrations that were 2:1 relative to serum and/or potassium levels 1.4:1, in conjunction with confirmed urine leakage.

To further assess the correlation between fluid characteristics and ultrasonographic appearance, abdominal fluid was also categorized according to the TNCC and TPC, respectively. The TNCC categories were low (< 1,500 cells/μL), medium (1,500–4,999 cells/μL), and high (≥5,000 cells/μL). The TPC groups were low: < 2.0 g/dL; medium: 2.4–4.5 g/dL; and high: >4.5 g/dL (1–3).

Ultrasonographic evidence of peritoneal fluid was confirmed by static images and/or cinematic loops obtained during the examinations. These assessments were conducted using an iU22 ultrasound system (Philips Medical System) or an RS85 Prestige ultrasound system (Samsung Medical System), both of which were equipped with a curvilinear probe operating within a frequency range of 5–10 MHz. System settings, including overall gain, time-gain compensation (TGC), dynamic range, focal zone placement and depth, were carefully optimized for each patient, as the visibility of small echogenic foci within the peritoneal fluid is highly dependent on these parameters. The scans and abdominocentesis were performed by a board-certified veterinary radiologist or a supervised resident. The positioning of the animals during the scans was based on the sonographer's preference. A ECVDI-certified veterinary radiologist reviewed the quality of the sonographic images, and any cases deemed to have insufficient quality images were excluded from the study. In the current investigation, a single board-certified veterinary radiologist and a diagnostic imaging resident, both unaware of the dogs' clinical histories and laboratory findings, conducted independent assessments of the archived static DICOM images and cine loops of the peritoneal effusion. The echogenicity of peritoneal effusions was categorized as either anechogenic or echogenic based on the overall appearance and the number of discrete echogenic foci observed. Fluids that appeared uniformly anechogenic, or that contained only a small amount of echogenic foci in the far field, were classified as anechogenic. Fluid with a larger amount or confluent echogenic foci were classified as echogenic. In instances of disagreement between the radiologist's and the resident's assessments, the relevant images were re-evaluated collaboratively, in a blinded fashion, until a consensus regarding the echogenicity of the peritoneal fluid was achieved for all dogs.

Statistical analyses were performed using SPSS Statistics for Macintosh, version 25.0 (IBM Corp., Armonk, NY, USA). A Pearson chi-square test using a 2 × 2 contingency table was used to assess the association between echogenicity and exudative vs. transudative classification. Pearson chi-square tests on 2 × 3 contingency tables were used to assess associations between echogenicity and TNCC and TPC categories. Fisher's exact test was used to compare septic and non-septic exudates because one cell contained zero observations. Odds ratios (ORs) and 95% confidence intervals (CIs) were calculated when estimable. Sensitivity, specificity, positive predictive value (PPV), and negative predictive value (NPV) were calculated using echogenicity as a positive test for exudate or “other” effusions and anechogenic appearance as a negative test. Statistical significance was set at *p* < 0.05.

## Results

The 100 dogs with peritoneal effusion included in this study represent a diverse cohort in terms of age, breed, and gender. The age of the dogs ranged from 13 weeks to 13 years (mean 6.6 years). The most commonly observed dog breeds in the study were Cross Breed (14 dogs), Labrador Retrievers (13 dogs), and Cocker Spaniels (nine dogs). Other notable breeds included Greyhounds (five dogs) and Jack Russell Terriers (four dogs), along with a variety of additional purebred and mixed-breed dogs (55 dogs) contributing to the overall findings. The dataset included 34 neutered males, 47 neutered females, 14 entire males, five entire females.

Following evaluation of the clinical history, cytological findings, laboratory results, imaging findings, and final diagnosis, the conditions associated with peritoneal effusion were grouped into the following etiological categories: neoplasia in 31 dogs, inflammatory disease in 30 dogs, septic peritonitis in 17 dogs, bile peritonitis in six dogs, conditions associated with increased hydrostatic pressure in eight dogs, and miscellaneous conditions in eight dogs. Peritoneal effusions were classified into three groups: exudate, transudate, and other. Exudates were identified in 40 dogs, transudates in 41 dogs, and 19 dogs were included in the “other” category (Table 1).

**Table 1:** Etiologies and fluid classifications of peritoneal effusions in 100 dogs.

Etiology	Disease	Exudate	Transudate	Other
Neoplasia (31)	Ruptured splenic hemangiosarcoma (6)	0	0	6
	Ruptured hepatic hemangiosarcoma (2)	0	0	2
	Hepatic carcinoma (3)	0	0	3
	Hepatic cystadenocarcinoma (1)	1	0	0
	Alimentary lymphoma (5)	5	0	0
	Multicentric lymphoma (4)	2	2	0
	Hepatic lymphoma (1)	0	1	0
	Metastatic apocrine anal sac adenocarcinoma (2)	2	0	0
	Adrenal tumor invading the caudal vena cava (1)	0	1	0
	Heart-base tumor (1)	1	0	0
	Intestinal carcinoma (2)	2	0	0
	Urothelial cell carcinoma (1)	0	0	1
	Histiocytic sarcoma (1)	1	0	0
	Neuroendocrine peritoneal mass (1)	0	0	1
	Subtotal	14	4	13
Inflammation (30)	Protein-losing enteropathy (5)	0	5	0
	Pancreatitis (10)	4	6	0
	Acute gastroenteritis (2)	0	2	0
	Haemorrhagic enteritis (1)	0	1	0
	Ulcerative duodenitis (1)	0	1	0
	Mesenteric volvulus (1)	1	0	0
	Jejunal abscess (2)	2	0	0
	Intestinal foreign-body obstruction (2)	1	1	0
	Cholecystitis (2)	1	1	0
	Hepatitis/cholangiohepatitis (4)	0	4	0
	Subtotal	9	21	0
Septic peritonitis (17)	Gastrointestinal rupture (17)	17	0	0
Bile peritonitis (6)	Gallbladder rupture (6)	0	0	6
Increased hydrostatic pressure (8)	Right-sided congestive heart failure (4)	0	4	0
	Chronic hepatopathy and portal hypertension (4)	0	4	0
	Subtotal	0	8	0
Miscellaneous (8)	Immune-mediated thrombocytopenia (2)	0	2	0
	Immune-mediated hemolytic anemia (2)	0	2	0
	Acute kidney injury (1)	0	1	0
	Road traffic accident (1)	0	1	0
	Protein-losing nephropathy (1)	0	1	0
	Steroid-responsive meningitis-arteritis (1)	0	1	0
	Subtotal	0	8	0
Total		40	41	19

Values represent numbers of dogs.

Within the exudate group, 17 of 40 dogs (43%) had septic effusions and 23 (57%) had non-septic effusions. Within the transudate group, 25 of 41 dogs (61%) had protein-poor effusions and 16 (39%) had protein-rich effusions. The “other” group comprised 12 dogs with hemoperitoneum, six with bile peritonitis, and one with uroperitoneum (Table 2, Supplement 2).

**Table 2:** Cytological classification of the peritoneal effusion and ultrasonographic appearance in 100 dogs.

Fluid type	Subtype	Echogenic	Anechogenic
Exudate (40)	Septic (17)	17	0
	Non-septic (23)	15	8
	Subtotal	32	8
Transudate (41)	Protein-poor (25)	2	23
	Protein-rich (16)	16	0
	Subtotal	18	23
Other (19)	Hemoperitoneum (12)	11	1
	Bile peritonitis (6)	5	1
	Uroperitoneum (1)	0	1
	Subtotal	16	3
Total		66	34

Supplement 2, numbers represent the number of dogs.

**Figure 1:**
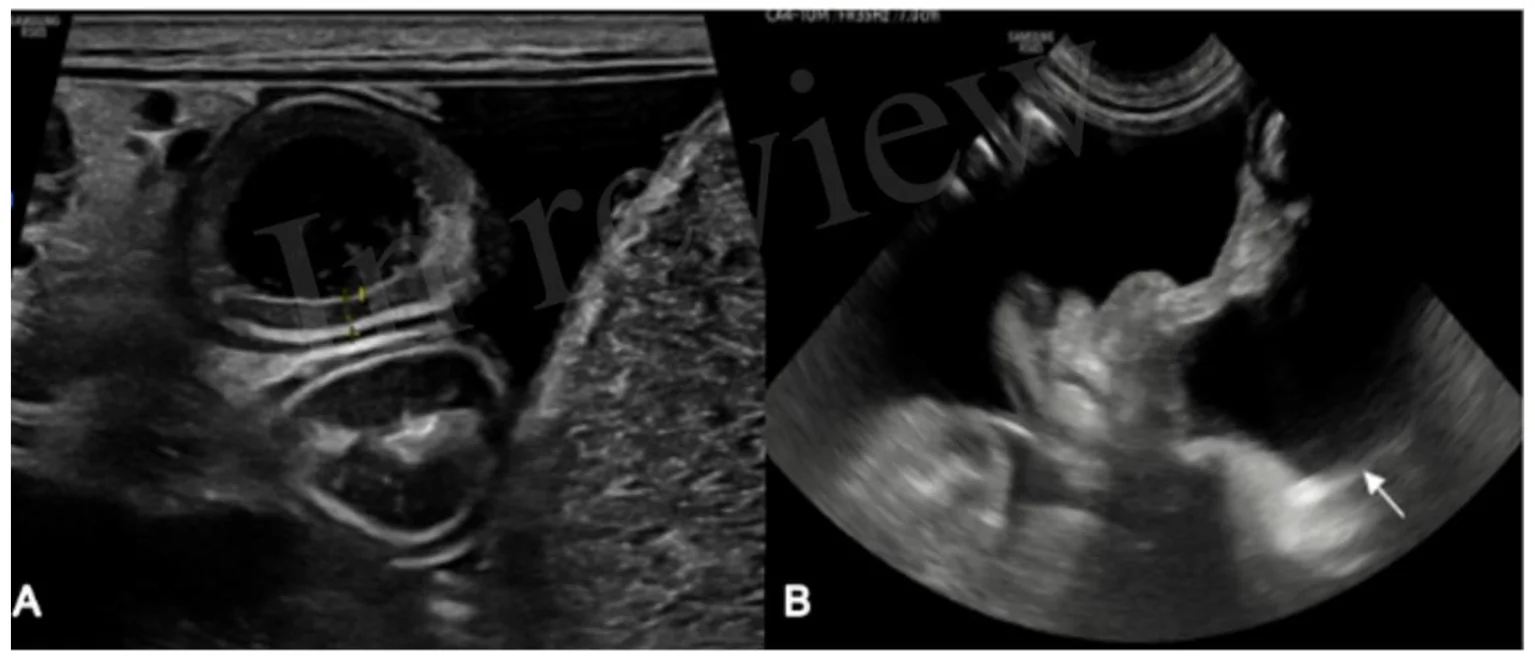
Anechogenic ascites. **(A)** Protein-poor transudate in a dog with protein losing enteropathy; **(B)** protein-rich transudate in a dog with obstruction of the CVC due to a left adrenal mass; note the few echogenic foci in the far field (arrow).

**Figure 2:**
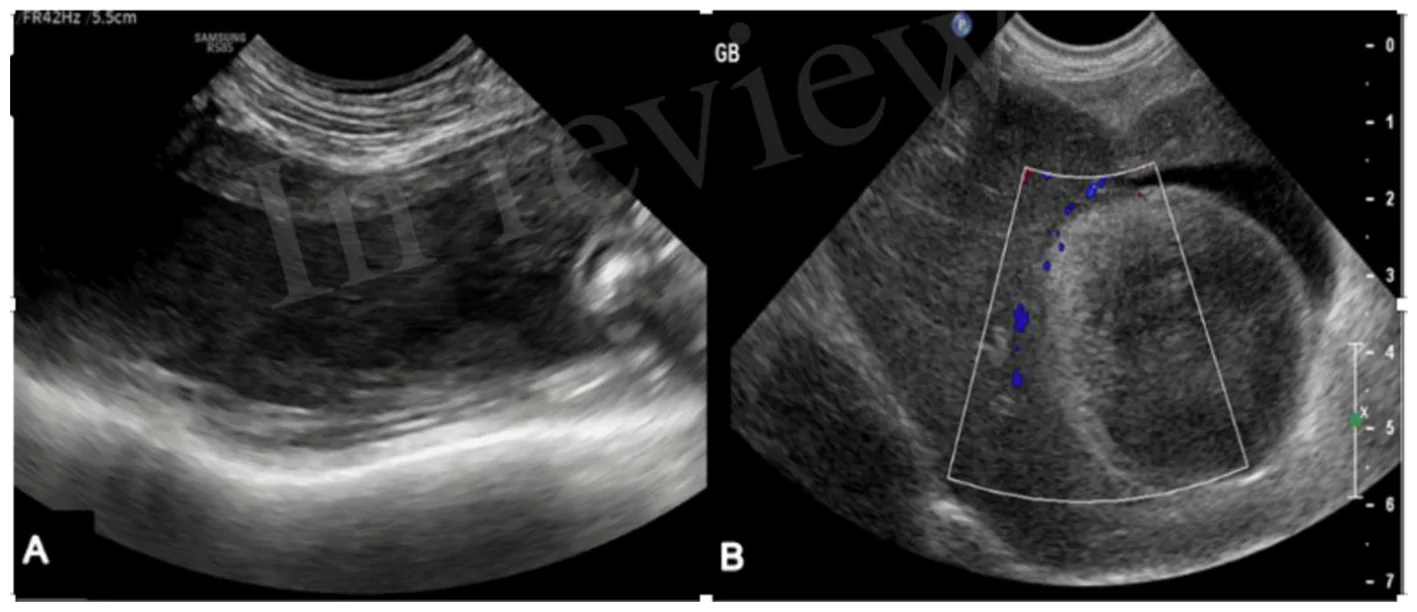
Echogenic ascites. **(A)** Hemorrhagic effusion in a dog with ruptured hemangiosarcoma; **(B)** bile peritonitis in a dog with ruptured mucocele.

Out of the 100 peritoneal effusions, 66 were classified as echogenic and 34 as anechogenic (Table 2). Among the 40 exudates, 32 (80%) were echogenic, whereas 18 out of 41 transudates (44%) were echogenic (Figures 1A, B). Out of 12 dogs with hemoperitoneum, 11 (92%) had an echogenic effusion. Ascites associated with bile peritonitis was echogenic in five out of six dogs (83%; Figures 2A, B). The single case of uroperitoneum was anechogenic.

A significant association was identified between ultrasonographic echogenicity and fluid classification when exudates were compared with transudates. Exudates were significantly more likely to appear echogenic than transudates (Pearson's χ^2^ = 11.17, df = 1, *p* < 0.001). The odds of an echogenic appearance were approximately five times greater for exudates than for transudates (OR = 5.11, 95% CI: 1.90–13.76; Table 2).

**Figure 3:**
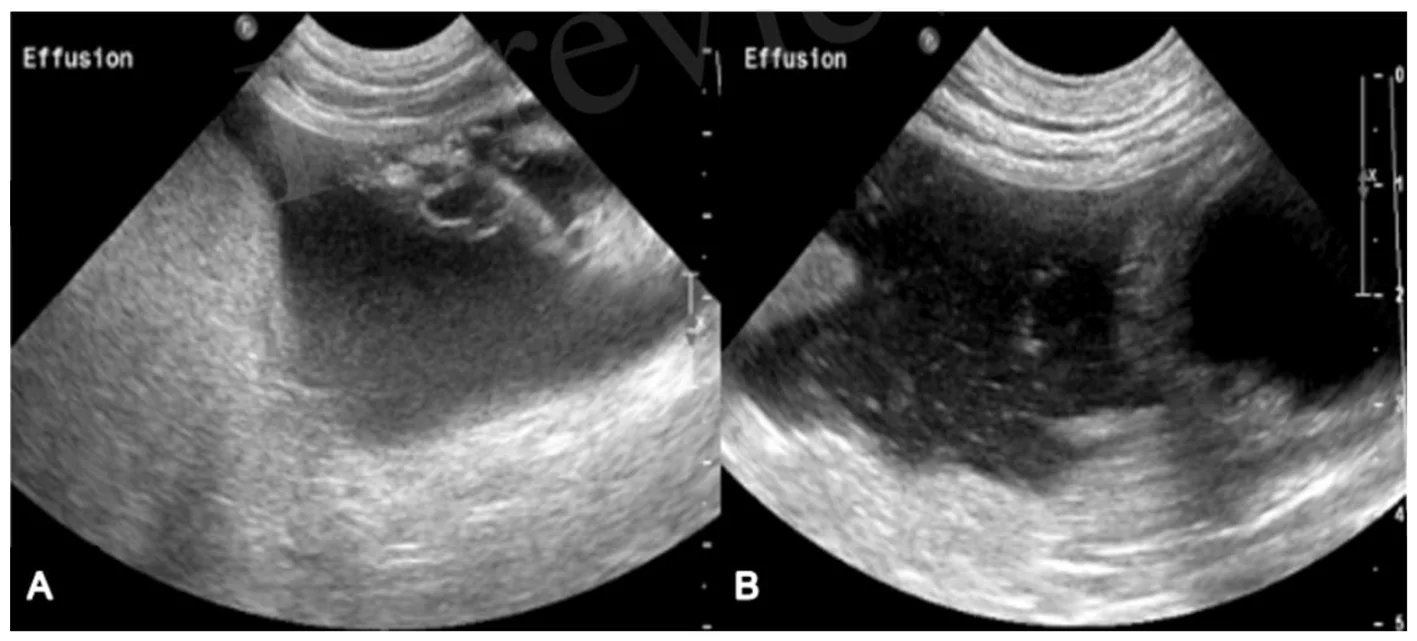
Echogenic ascites. **(A)** Non-septic exudate in a dog with multicentric lymphoma; **(B)** septic exudate in a dog with intestinal perforation due to an obstructing foreign body. Note the finely granular and uniform appearance of the peritoneal fluid in panel **(A)** and the coarser and more variable shape of the echogenic foci in panel **(B)**.

When septic and non-septic exudates were compared, both groups predominantly showed an echogenic appearance (Figures 3A, B). All 17 septic exudates (100%) were echogenic, compared with 15 out of 23 non-septic exudates (65%; Table 2).

The association between septic status and echogenic appearance was statistically significant (Fisher's exact test, *p* = 0.013). However, because of the zero value in one cell of the contingency table, a conventional odds ratio could not be reliably estimated. This result should therefore be interpreted cautiously, particularly given the relatively small number of septic effusions.

When echogenicity was used to identify exudates and effusions included in the “other” category, the sensitivity was 81%, specificity was 56%, positive predictive value was 73%, and negative predictive value was 68%.

Abdominal fluid was also categorized according to TNCC (Table 3, Supplement 3). All exudates had cell counts exceeding 5,000 cells/μL. Septic exudates had a higher mean TNCC, approximately 61,000 cells/μL, compared with approximately 28,000 cells/μL in non-septic exudates, indicating a greater inflammatory burden in septic effusions.

**Table 3:** Echogenicity of peritoneal fluid and total nucleated cell count (TNCC) values.

Ultrasonographic appearance	Low TNCC (< 1,500/μL)	Medium TNCC (1,500–4,999/μL)	High TNCC (≥5,000/μL)	Total
Echogenic	6	10	50	66
Anechogenic	26	3	5	34
**Total**	32	13	55	100

Supplement 3, numbers represent the number of dogs.

The distribution of peritoneal fluid echogenicity across the three TNCC groups is summarized in Table 3. Notably, 50 out of 66 dogs (76%) with echogenic peritoneal fluid were classified in the high-TNCC group. Conversely, 26 out of 34 dogs (76%) with anechogenic fluid were classified in the low-TNCC group. A significant association was identified between TNCC category and ultrasonographic appearance (Pearson's χ^2^ = 47.74, df = 2, *p* < 0.001), indicating that effusions with higher nucleated cell counts were more likely to appear echogenic.

The echogenicity of peritoneal fluid across the three TPC categories is summarized in Table 4, Supplement 4. Among the 66 dogs with echogenic effusion, 19 (29%) were in the low-TPC group, 32 (48%) in the medium-TPC group, and 15 (23%) in the high-TPC group. Among the 34 dogs with anechogenic fluid, 19 (56%) were in the low-TPC group, 11 (32%) in the medium-TPC group, and 4 (12%) in the high-TPC group. A significant association was identified between TPC category and ultrasonographic appearance (Pearson's χ^2^ = 7.11, df = 2, *p* = 0.029), indicating that fluids with higher protein concentrations were more likely to appear echogenic on ultrasonography. All the statistical analyses performed are summarized in Table 5.

**Table 4:** Echogenicity of peritoneal fluid and total protein concentration (TPC) values.

Ultrasonographic appearance	Low TPC (< 2.0 g/dL)	Medium TPC (2.4–4.5 g/dL)	High TPC (>4.5 g/dL)	Total
Echogenic	19	32	15	66
Anechoic	19	11	4	34
Total	38	43	19	100

Supplement 4, numbers represent the number of dogs.

**Table 5:** summary of statistical analysis and interpretation.

Analysis	Groups compared	Statistical test	Results	Interpretation
Echogenicity vs. fluid classification	Exudate vs. transudate	Pearson's chi-square	χ^2^ = 11.17, df = 1, *p* < 0.001	Exudates were significantly more likely to be echogenic than transudates
Odds of echogenicity	Exudate vs. transudate	Odds ratio	OR = 5.11, 95% CI: 1.90–13.76	Odds of echogenicity were approximately five times higher in exudates
Septic vs. non-septic exudate	Septic vs. non-septic exudates	Fisher's exact test	*p* = 0.013	Septic exudates were significantly more often echogenic
Odds ratio for septic status	Septic vs. non-septic exudates	Not reliably estimable	Not reported	One cell contained zero anechogenic septic effusions
Diagnostic performance	Echogenicity for exudate/“other” vs. transudate	Diagnostic indices	Sensitivity 81%; specificity 56%; PPV 73%; NPV 68%	Echogenicity was sensitive but only moderately specific
Echogenicity vs. TNCC category	Low, medium, high TNCC	Pearson's chi-square	χ^2^ = 47.74, df = 2, *p* < 0.001	Higher TNCC was strongly associated with echogenicity
Echogenicity vs. TPC category	Low, medium, high TPC	Pearson's chi-square	χ^2^ = 7.11, df = 2, *p* = 0.029	Higher TPC was significantly associated with echogenicity

TNCC, total nucleated cell count; TPC, total protein concentration; OR, odds ratio; CI, confidence interval; PPV, positive predictive value; NPV, negative predictive value.

## Discussion

The study demonstrated a significant association between the ultrasonographic echogenicity of peritoneal effusion and fluid classification in dogs. Exudates were substantially more likely than transudates to appear echogenic, and the odds of an echogenic appearance were approximately five times greater for exudates than for transudates. Echogenicity was also strongly associated with increasing TNCC and was significantly associated with TPC category. These findings support the initial hypothesis that exudative effusions are more likely to appear echogenic because of their greater cellular and protein content. The diagnostic performance results indicate that echogenicity may be useful as a rapid screening observation but is not sufficiently accurate to replace fluid analysis. When echogenicity was used to identify exudate or “other” effusions, sensitivity was 81%, whereas specificity was 56%. Thus, an echogenic appearance increased suspicion for a cellular, protein-rich, hemorrhagic, or biliary effusion, but a substantial proportion of transudates were also echogenic. Similarly, an anechogenic appearance reduced but did not eliminate the possibility of an exudate or other clinically important effusion. Certain inflammatory conditions, such as pancreatitis or cholecystitis, as well as acute hemorrhage, may initially present with anechogenic peritoneal fluid. However, as these conditions progress, the peritoneal fluid may become more echogenic.

The strong association between echogenicity and TNCC was the most pronounced statistical relationship in the study. Most echogenic effusions were in the high-TNCC category, whereas most anechogenic effusions were in the low-TNCC category. The association with TPC was weaker but remained statistically significant. These findings suggest that the ultrasonographic appearance of peritoneal fluid is influenced by its particulate content, including cells, proteins, fibrin, cellular debris, and blood products. Rather than representing a characteristic of a single disease or fluid type, echogenicity therefore appears to reflect the overall cellular and biochemical composition of the effusion.

Both septic and non-septic exudates predominantly appeared echogenic. All 17 septic exudates were echogenic, compared with 15 of 23 non-septic exudates, and this difference was statistically significant. This finding is clinically relevant because septic peritonitis may require urgent surgical and antimicrobial management. However, this result should be interpreted cautiously because the septic group was relatively small and no septic effusion was anechogenic, resulting in a zero cell in the contingency table and preventing reliable estimation of a conventional odds ratio. Echogenicity should therefore be considered an adjunctive finding rather than a diagnostic criterion for sepsis. Cytology, culture where appropriate, biochemical analysis, imaging findings, and the overall clinical context remain essential (12).

Moreover, although septic effusions had a higher mean TNCC than non-septic exudates, the difference in TNCC should not be interpreted as a sufficiently specific discriminator of sepsis. Previous veterinary research has similarly reported higher TNCC, neutrophil counts, and protein concentrations in septic than in non-septic peritoneal effusions, reflecting a greater inflammatory burden (13). Nevertheless, considerable overlap may occur, and neither echogenicity nor TNCC can replace cytological identification of intracellular bacteria or other laboratory evidence of sepsis. Assessment of echogenicity may provide useful preliminary information during abdominal ultrasonography, particularly in emergency situations or when immediate laboratory assessment is unavailable. The result of our study showed that ultrasonography is useful for characterizing peritoneal fluid echogenicity and, by extension, narrowing down underlying causes. An echogenic effusion increases suspicion of an exudative, hemorrhagic, biliary, or otherwise particle-rich effusion, but it is not sufficiently specific to establish a diagnosis.

The distribution of disease categories in the present population further illustrates this overlap. Effusions associated with low cellularity or lower protein concentrations, including many transudates related to protein-losing or hydrostatic disorders, were commonly anechogenic or only mildly echogenic. In contrast, septic, hemorrhagic, biliary, and several neoplastic effusions frequently appeared echogenic. However, inflammatory and neoplastic diseases did not produce uniform ultrasonographic appearances. These findings reinforce that echogenicity reflects fluid composition rather than a specific underlying etiology and should not be used to assign a disease category without corroborating evidence. Echogenicity should therefore be interpreted alongside the distribution and volume of fluid, associated abdominal abnormalities, clinical history, hematological and biochemical findings, and fluid analysis (12).

The present findings are broadly consistent with human imaging literature, in which echogenic ascitic fluid has been associated with exudative, hemorrhagic, malignant, or inflammatory causes (9–12, 14–16). However, previous studies have also cautioned that sonographic appearance alone cannot reliably establish fluid composition or etiology (11, 12, 14–16). This relationship has been well documented in previous research assessing the role of various imaging modalities in differentiating transudative from exudative ascites (11, 12).

Previous studies in humans investigating ultrasonography and CT have also demonstrated that imaging characteristics may assist in differentiating transudative, exudative, benign, and malignant ascites, although substantial overlap remains (11, 12, 14–16). CT can provide quantitative attenuation measurements and has shown higher attenuation values in some malignant or protein-rich effusions. However, ultrasonography offers practical advantages in veterinary patients because it is widely available, relatively inexpensive, does not involve ionizing radiation, permits real-time assessment of fluid movement, and can be used to guide abdominocentesis (9–11).

Previous ultrasonographic descriptions have proposed more detailed patterns of fluid echogenicity, including: flocculent echogenicity that may indicate a more cellular effusion, septate patterns that could be associated with peritonitis or chronic and granulomatous conditions, clotted echogenicity that might suggest hemorrhage, speckled patterns that could signify protein-rich fluid, and whirling echogenicity that may be linked to increased cell numbers, inflammation, or active bleeding (6–8). These patterns may correspond to differences in cellularity, fibrin content, chronicity, hemorrhage, or active fluid movement.

The current study intentionally used a simpler binary classification of echogenic vs. anechogenic fluid to improve clinical applicability and accommodate the retrospective nature of the available images. Although this approach identified significant associations with fluid classification, TNCC, and TPC, it may have reduced the ability to distinguish more subtle or potentially diagnostically relevant patterns.

Several limitations should be considered when interpreting these findings. The retrospective design restricted control over image acquisition, patient positioning, ultrasound settings, and the number and location of images obtained. Echogenicity is influenced by overall gain, time-gain compensation, dynamic range, focal-zone placement, depth, transducer frequency, and the amount of fluid present. Although image quality was reviewed and inadequate studies were excluded, variation in acquisition parameters may have affected the visibility of small echogenic foci.

Evaluation was based predominantly on archived static images, with cine loops available for only a proportion of cases. Static images may not demonstrate dynamic features such as swirling cells, mobile debris, fibrin strands, or settling blood products. Patient positioning may also influence the distribution of particulate material, potentially causing echogenic contents to settle outside the imaged region. The binary assessment of echogenicity was subjective. Although images were independently reviewed by two observers and disagreements were resolved by consensus, interobserver agreement was not formally calculated. Some subgroups, particularly septic exudates, bile peritonitis, and uroperitoneum, contained relatively few cases. The diagnostic performance estimates and subgroup comparisons should therefore be interpreted cautiously. Finally, the study population was obtained from a single referral hospital, which may limit the generalizability of the findings to primary-care populations.

Future prospective studies should use standardized acquisition protocols, including consistent transducer frequency, gain, dynamic range, focal-zone placement, patient position, and image location (9–11). Both static images and cine loops should be obtained to assess dynamic fluid characteristics. Evaluation by multiple blinded observers would allow calculation of intra- and interobserver agreement. Larger, multicenter populations would also permit more reliable analysis of individual effusion types and underlying disease categories.

Further investigation of quantitative approaches may reduce the subjectivity of visual assessment. B-mode gray-scale histogram analysis, which has been explored in human medicine, could provide an objective measurement of fluid echogenicity (11). Standardized assessment of specific echogenic patterns, such as homogeneous fine echoes, coarse particulate material, fibrin strands, septations, clots, or swirling debris, may also improve diagnostic discrimination. Comparative studies between ultrasonography, CT attenuation, cytology, TNCC, TPC, and fluid-specific biochemical markers could determine whether combining imaging and laboratory variables produces more accurate predictive models than echogenicity alone (12, 17, 18).

In conclusion, echogenicity was significantly associated with peritoneal-fluid classification, TNCC, and TPC in dogs. Exudates were more likely to be echogenic than transudates, and all septic exudates in this population had an echogenic appearance. Our results show that it is unlikely for an exudate to appear anechogenic, and that septic effusions are highly likely to appear echogenic. However, the moderate specificity and overlap among fluid categories preclude the use of echogenicity as an independent diagnostic test. Assessment of echogenicity may provide rapid, clinically useful preliminary information, but it should complement, rather than replace, cytological and biochemical fluid analysis. Cytological identification of intracellular bacteria, supported by appropriate biochemical and microbiological testing, remains essential when septic peritonitis is suspected (18).

## Data Availability

The raw data supporting the conclusions of this article will be made available by the authors, without undue reservation.
